# Correspondence as to the Treatment of Burns by White Lead

**Published:** 1876-12

**Authors:** 


*Dr. R. W. Westmoreland:*

Dear Doctor—If not inconvenient, please state, either through the columns of the Journal or privately to myself, your mode of applying the white lead paint and cotton pads to burns? Also, do you use and expect benefit from it in recent burns? I mean as a first application. I am deeply interested in finding a remedy which is *good* and convenient to apply for this class of injuries, as I always have trouble in treating them successfully. As an earnest well-wisher to the JOURNAL and yourself, I am, Yours truly,

JNO. G. MCKENNY.

*Hickory Grove, Ga., Dec.* 8, 1876.

The foregoing correspondence has just been received, and we take this means of replying.

Dr. Gross, so far as we are informed, first brought the treatment of burns by white lead prominently into notice. We have tried it often with most happy results. To the preparation of the pure white lead, as used by painters, we add a sufficient quantity of linseed oil to give it the proper consistency, and then paint it on the burn thoroughly. Over this place cotton pads, carded into a state of sufficient softness, and over all sew or pin a soft cloth for protection.

We have applied this in very recent, as well as those burns which had existed for some time, and always with speedy relief and good cure. The *modus operandi* of the remedy is found probably in the sedative effect of the lead, as well as in the protection to the denuded surface afforded by the impervious coating of paint and cotton.

R. W. WESTMORELAND.

